# 

**Published:** 1887-12

**Authors:** 


### TABLE OF CONTENTS.

ORIGINAL AND SELECTED ARTICLES.

The Nervous Phenomena Observed in a Case of Exposure of the Anterior Column of the Cord from Syphlitic Ulceration of Upper Pharynx, by A. G. Hobbs, M.D., Atlanta, Ga. 486
Incipient Insanity, by H. E. Stroud, M.D., Grand Junction, Col. 488
Melon Root 489
Bichloride of Mercury as a Germicide, by H. E. Stroud, M D., Grand Junction, Col 490
Cocaine in Croup 491
Recent Studies of Hypnotism 492
Is Pulmonary Consumption Contagious, by A. Brochin, M.D 494
The Treatment of Corpulency, by Ross R. Bunting, M.D 497
When Should the Ovaries be Removed, by William Goodell, M.D 498

ABSTRACTS AND GLEANINGS.

Antipyrine 501
The Indications and Rationale in Washing Out the Puerperal Uterus 502
Positions that Affect Sleep 503
Experiments with Antifebrin; The Treatment of Chronic Abscess by Injections of an Ethereal Solution of Iodoform 504
A Case of Traumatic Tetanus Cured 505
Hydroquinone; Boracic Acid Packing in Leucorrhœa 506
Salicylate of Ammonium as an Antipyretic; Treatment of Ruptured and Divided Tendons 507
Treatment of Otorrhœa; A New Hypnotic; Instantaneous Cure of Rheumatical Lumbago. 508
Bromide of Ethyl; Antipyretics; Eucalyptus in Whooping-Cough and Bronchitis; Effect of Carbolic Acid on Temperature 509
Antipyrine Hypodermically as a Substitute for Morphine; The Toxical Properties of Cocaine; A Solvent for Antifebrin 510
Antipyrine, Hypodermically, instead of Morphine; Ergot Again; Fowler's Solution for Nervous Condition of Menopause 511
Ergot in Ophthalmic Practice 512
Typhoid Fever; How to Preserve the Dead Body; Cocaine Internally 513
Castration of Criminals; New Remedies for the Morphine Habit; Succedaneum of Morphia; Seven Consecutive Cases of Charbon Treated Successfully by Excision; Eucalyptus Honey; Quinine 514

SCIENTIFIC ITEMS.

Electrical Ice Cream Poison; Vest Button Photography 515
Arctic Cold; Home-made Ice 516

PRACTICAL NOTES AND FORMULÆ.

Prurigo; Local Anæsthetic; Instantaneous Remedy for Lumbago 517
Ointment for Scabies; For Consumption; Antiseptic Powder; Solution of Antifebrin; Mixture for Flatulent Dyspepsia; A Mixture for Infantile Diarrhœa 518
An Anodyne for Dental Caries; To Prevent Waste of Tissue; Treatment of Diabetes; For Bronchitis; For Pharyngitis 519

EDITORIALS AND MISCELLANEOUS.

Editorial Brevities; Our Friends Must Write 520
To Our Readers; Types of Fever 521
A Query 522
Gaston's Operation; Professor Charcot; Books and Pamphlets Received 524
Receipted; Prof. Loisette's Memory Discovery; Special Notes 527

